# Harnessing data science to advance radiation oncology

**DOI:** 10.1002/1878-0261.12685

**Published:** 2020-05-18

**Authors:** Ivan R. Vogelius, Jens Petersen, Søren M. Bentzen

**Affiliations:** ^1^ Deptartment of Oncology Rigshospitalet Copenhagen Denmark; ^2^ Faculty of Health and Medical Sciences University of Copenhagen Denmark; ^3^ Deptartment of Computer Science University of Copenhagen Denmark; ^4^ Department of Epidemiology & Public Health Greenebaum Cancer Center University of Maryland Baltimore MD USA

**Keywords:** artificial intelligence, data science, radiotherapy

## Abstract

Radiation oncology, a major treatment modality in the care of patients with malignant disease, is a technology‐ and computer‐intensive medical specialty. As such, it should lend itself ideally to data science methods, where computer science, statistics, and clinical knowledge are combined to advance state‐of‐the‐art care. Nevertheless, data science methods in radiation oncology research are still in their infancy and successful applications leading to improved patient care remain scarce. Here, we discuss data interoperability issues within and across organizational boundaries that hamper the introduction of big data and data science techniques in radiation oncology. At the semantic level, creating common underlying models and codification of the data, including the use of data elements with standardized definitions, an ontology, remains a work in progress. Methodological issues in data science and in the use of large population‐based health data registries are identified. We show that data science methods and big data cannot replace randomized clinical trials in comparative effectiveness research by reviewing a series of instances where the outcomes of big data analyses and randomized trials are at odds. We also discuss the modern wave of machine learning and artificial intelligence as represented by deep learning and convolutional neural networks. Finally, we identify promising research avenues and remain optimistic that the data sources in radiation oncology can be linked to yield important insights in the near future. We argue that data science will be a valuable complement to, but not a replacement of, the traditional hypothesis‐driven translational research chain and the randomized clinical trials that form the backbone of evidence‐based medicine.

## Graphical abstract

Data science has an important role to play in the technology and computer‐intensive field of radiation oncology, but requires multidisciplanry teams and domain knowledge to be successful. We argue that data science will be a valuable complement to, but not a replacement of, the traditional hypothesis‐driven translational research chain and the randomized clinical trials.

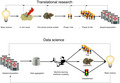

**Abbreviations**
API: application programming interface
DL: deep learning
EHR: electronic health record
PRO: patient‐reported outcome
R&V: electronic record‐and‐verify systems
RGC: Radiogenomics Consortium
SNP: single nucleotide polymorphism

## 1 Introduction

Data science is a multidisciplinary field that uses scientific methods, processes, algorithms, and systems to extract knowledge and insights from structured and unstructured data (Wikipedia, 2019). Data science is emerging as a new paradigm in the biomedical sciences, distinct from conventional theoretical and empirical science. In this new paradigm, patterns detected in large sets of clinical data provide a means to understand the nature of disease and its response to therapy, either alone or by representing a bedside‐to‐bench inverse translation, in which hypotheses are derived from clinical outcome data and then later studied in detail in the laboratory or tested in controlled clinical trials.

A successful data science project should combine computer science, statistical knowledge, and domain knowledge from the field of interest.

Radiotherapy is a mainstay in modern anticancer therapy and is indicated in more than half of cancer patients at some point during disease management (Lievens and Grau, 2012). Modern radiotherapy is a computer‐intensive and technology‐heavy discipline with regulatory requirements for documenting and verifying radiotherapy exposure and should therefore be well suited for data science advances.

**Fig. 1:**
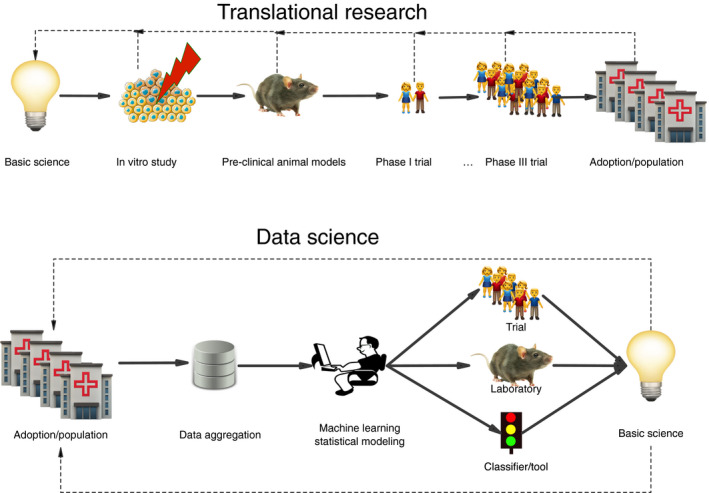
The translational research chain versus the data science approach.

In the present review, we focus on data science in radiation oncology, as defined by the combination of data analytics and big data sources with the ultimate aim of gaining insights into improved therapy for future cancer patients. The data science paradigm can be contrasted with the translational research chain paradigm (Fig. 1), and in this review, we define data science as shown in Fig. 1. It should be mentioned that the workflow in radiation oncology is complex and involves many tasks that are likely to be successfully automated or at least partly supported by tools developed using machine learning techniques (Meyer *et al.*, 2018; Thompson *et al.*, 2018). Although there are some promising parallels between these efforts and the data science approach discussed here, it is beyond the scope of this review to cover the automation of radiation treatment planning and delivery in any detail.

Data science is, in many ways, a compelling concept in the field of radiation oncology. First and foremost, a data science approach seeks to analyze data on human patients treated as part of clinical routine. Preclinical tumor models show many important biological differences to spontaneous tumors in humans. With current advances in the understanding of the importance of host–disease interactions (Hanahan and Weinberg, 2011), in particular with the rise of immuno‐oncology, including immunotherapy–radiotherapy combinations, the limitations of relying mainly on *in vitro* assays and small‐animal models to develop new therapies are clear. Secondly, adverse events of radiation therapy depend not only on the detailed deposition of dose in time and space but also on patient‐level comorbidities, comedications, and patient age (Bentzen, 2006). These are cofactors that are difficult to represent adequately in preclinical models. Also, the analysis of routine clinical data is a promising complement. Indeed, we have seen examples where human outcome data analysis has been used to describe the detrimental impact of radiotherapy on the immune system (Shiraishi *et al.*, 2018; Terrones‐Campos *et al.*, 2019; van Rossum *et al.*, 2020). Such findings might may serve as an important context for preclinical studies that aim to explore the beneficial effect of radiation for immune therapy (Durante and Formenti, 2019; Formenti and Demaria, 2013). Thirdly, although the controlled clinical trial is very likely to remain as the gold standard in evidence‐based radiation oncology (Bentzen, 1998; Bentzen and Yarnold, 2014), there are numerous types of questions that cannot easily be subjected to clinical trial methodology. Big data from population‐level registries are increasingly emerging as an important complement to trial outcomes, as discussed further below. In the case of radiation oncology, however, the big data are unfortunately often not all that big.

In this review, we discuss the data sources involved in radiation oncology data science, the methodological hurdles to consider when using big data sources, and potential solutions and promising future research avenues. We finish with an important discussion of the clinical utility of the knowledge obtained from data science in radiation oncology.

## 2 Data sources and missing links in radiation oncology

Large databases of treatment and outcome data have long been available for healthcare providers and researchers in multiple countries and regions across the globe. Databases provide essential reimbursement mechanisms for healthcare providers across public and insurance‐based systems alike. Furthermore, most developed countries have national cancer registries with mandatory reporting of incident cases.

**Fig. 2:**
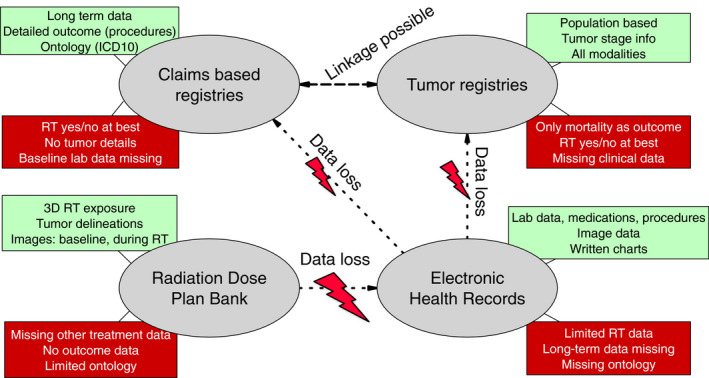
Key sources of data and points of radiation data loss. From lower left and counterclockwise, radiation dose plan banks are currently available in all modern institutions as record‐and‐verify systems that contain image data and 3D radiation dose exposure, but no follow‐up data. Hospital‐based EHR systems contain more detail on other treatments, but do not contain detailed, granular radiotherapy data; such data are often reduced to prescription dose/fractionation. Radiotherapy data are further lost when these data are moved to large claims‐based registries or tumor registries, where data on various aspects of long‐term outcome are available. Red boxes: examples of main shortcomings of data source. Green boxes: examples of main strengths.

At the hospital level, electronic health records (EHRs) have long been in routine use in developed countries, and few will disagree that EHRs will expand their future role as an integral part of the hospital data infrastructure. Finally, focusing on the radiotherapy providers, electronic record‐and‐verify (R&V) systems are mandatory for documenting detailed radiation exposure and for improving patient safety. Detailed exposure data for all patients are stored and must be retrievable for clinical use in the common event of a future indication for re‐irradiation. These different data sources, together with their key strengths and weaknesses, are shown in Fig. 2.

**Fig. 3:**
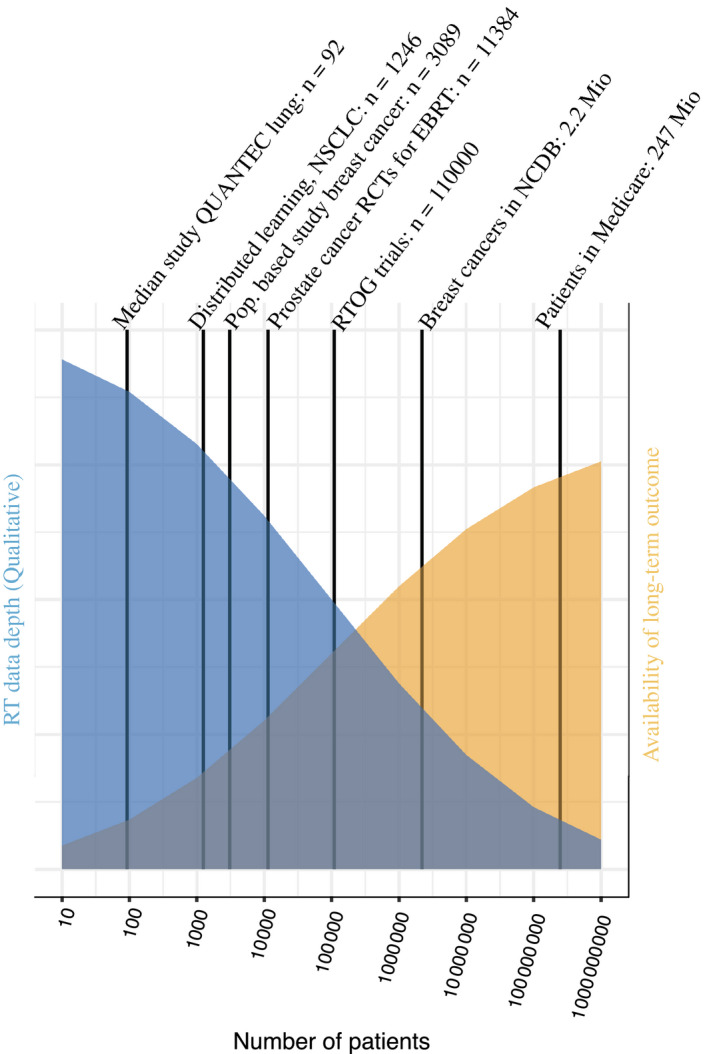
Schematic illustration of loss of granularity of radiotherapy data when moving from single institutional series to the largest of available datasets. This graph provides a schematic illustration of the level of radiotherapy data granularity versus sample sizes across selected published studies and available databases in the United States as examples. QUANTEC (Marks *et al.*, 2010a) is an example of a federated learning model that aims to bridge institutional series (Defraene *et al.*, 2019). Also shown is a population‐based series of breast cancer patients without detailed dosimetry, but with information available about whether internal mammary nodes were included in the radiotherapy target (Thorsen *et al.*, 2016). The graph also shows the number of patients in randomized trials of external beam radiotherapy (EBRT) for prostate cancer (Vogelius and Bentzen, 2018; Widmark *et al.*, 2019), the number of patients in Radiation Therapy Oncology Group (RTOG) trial databases (personal communication), and the number of breast cancer patients in the National Cancer Database (NCDB) and Medicare. Where long‐term outcomes are available in the large series (to the right), radiotherapy information is often reduced to one bit of information (radiotherapy given or not) in these studies (McGale *et al.*, 2016). Abbreviations: QUANTEC: Quantitative Analyses of Normal tissue Effects in the Clinic. RT, radiotherapy; RCT, randomized controlled trial.

We note a substantial challenge in radiotherapy data science: the immediate loss of detailed radiotherapy exposure data when moving data from the departmental R&V database to other data sources. Hospital EHRs will typically carry information on prescription dose, time, and delivered number of fractions, but not the 3D dose distribution data available from R&V records. When moving beyond the hospital to claims‐based registries or tumor registries, radiotherapy is often only recorded as a one‐bit yes/no item, which, with few exceptions, preclude meaningful inference with regard to the optimal use of radiotherapy. Conversely, the R&V databases lack the crucial long‐term outcome data from the larger systems (Fig. 3) and will often be limited in terms of the number of individuals treated. Even when patient‐level links exist between these databases, developing an ontology for defining structures and for reporting doses for R&V records, and for defining specific long‐term outcomes on the registry side, remains work in progress.

Powerful examples of the value of establishing a widely adopted ontology include the TNM classification for staging of cancer, developed between 1943 and 1952 by P. Denoix at the Institute Gustave‐Roussy. This ontology was subsequently adopted by the Union for International Cancer Control (UICC), the International Federation of Gynecology and Obstetrics (FIGO), and the American Joint Committee for Cancer (AJCC) (UICC, 2017). Another example is the development of the Common Terminology Criteria for Adverse Events (CTCAE), which has been widely adopted by many single or multimodality cancer studies (Trotti *et al.*, 2003). In radiation oncology, there have also been commendable efforts to standardize organ delineation for radiation therapy planning and the corresponding nomenclature used in routine clinical care (Duane *et al.*, 2017; Landberg *et al.*, 2016; Offersen *et al.*, 2015).

Nevertheless, ensuring the interoperability of databases requires us to do more. We need to define a set of minimum data elements to record radiation modality and delivery technique, which would be an important step forward (Hayman *et al.*, 2019). However, the effort needed to achieve a full, meaningful annotation of complex 3D imaging and exposure data is substantial. It should be recognized that manual annotations are often inconsistent even within a department and that they are generally restricted to the normal structures necessary for treating the patient in question; further details needed for research cannot be expected to be reliably annotated in clinical routine databases. Having said that, the retrospective estimation of dosimetry might be possible in the absence of 3D dosimetry data in selected cases: For example, cardiac exposure after breast and lymphoma treatment can be reasonably reliably quantified from 2D portal images or treatment descriptors in historical series (Darby *et al.*, 2013; Maraldo *et al.*, 2015; van Nimwegen *et al.*, 2017). Modern radiotherapy planning is becoming increasingly individualized, however, and therefore, such retrospective dosimetry will, in most cases, be unreliable with current radiotherapy (Maraldo *et al.*, 2012). The studies of cardiac exposure demonstrate another main strength of data science: the potential ability to study relatively rare, high‐grade toxicity endpoints of high clinical relevance, such as major coronary events (Darby *et al.*, 2013). Most institutional series will not have enough power to resolve any relevant exposure–risk relationships for high‐grade toxicity events. Consequently, low‐grade clinical endpoints (such as low‐grade radiation pneumonitis for lung exposure; Marks *et al.*, 2010a; Marks *et al.*, 2010b) often form the basis for recommended dose constraints, even when the disease itself has a dismal prognosis.

As an alternative to using the registries mentioned here, multiple institutions have successfully combined data in population‐based studies (Thorsen *et al.*, 2016) in which the numbers of cases reach several thousands, but in which dosimetry data remain limited. Finally, the concept of federated learning has been proposed and demonstrated as a means by which to combine insights from institutional series without the logistical and legal complexities of sharing patient data (Defraene *et al.*, 2019; Jochems *et al.*, 2017). However, the numbers of patients and the dosimetric granularity offered by current published examples of federated learning remain limited, as shown in Fig. 3. We now leave the data source discussion to focus on challenging aspects of the data analysis itself.

## 3 Applying data science to radiation oncology: The methodological challenges

Here, we discuss the methodological challenges of applying data science to radiation oncology. It should be emphasized that the covariates, as well as the endpoints that are analyzed, should be defined by clinical relevance or scientific interest and not by availability. When analyzing and publishing the data science material, it is relevant to keep some terms and concepts in mind. The safest route to appease a peer reviewer is to argue that the objective of a data science publication is to elucidate associations in data. A limitation of this approach, however, is that the presence of associations rarely has any clinical relevance. The next step would be to perform prediction of outcome. Predictive models rely on one or more covariates that together give an (ideally) robust assessment of the probability of a given endpoint (Collins *et al.*, 2014, 2015). Note that the covariates need not be causally related to the endpoint in question to provide an accurate outcome prediction. The downside of predictive modeling without causal content is that the use of further refined radiotherapy to modify a dose distribution‐related covariate might fail to provide the expected clinical benefit if other associations are broken, for example, when moving from photon to proton therapy. Causal relationships are generally preferable to both association studies and predictions without causal inference, but inference methods rely on avoiding bias. Here, the most robust method is the randomized controlled trial, but other methods exist where randomization is not feasible (Pearl, 2009).

**Fig. 4:**
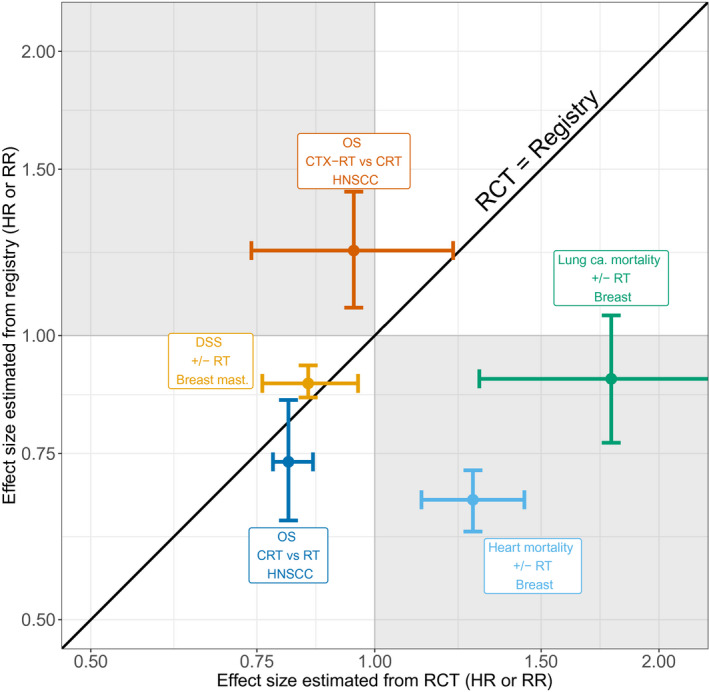
A comparison of effect‐size estimates from randomized controlled trials and registry‐based analyses. The schematic shows published effect‐size estimates from randomized controlled trials (*x*‐axis) and registry‐based analyses (*y*‐axis). Concordant effect sizes are indicated by the black identity line. We see examples of registry‐based studies over‐ and underestimating effects, as well as being relatively in agreement. Data are collected from Ang *et al. *(2014), McGale *et al. *(2016), Pignon *et al. *(2009), and Zandberg *et al. *(2018), and reanalyzed/replotted for this analysis by SMB and IRV. CRT, chemoradiotherapy; CTX‐RT, cetuximab + RT; DSS, disease‐specific survival; HNSCC, head‐and‐neck squamous cell cancer; OS, overall survival.

Although causal inference is challenging, and prone to residual bias and confounding even when exercising the best possible care (cf. Fig. 4), such methods are likely to be more efficient when combined with improved, prospective outcome reporting, across a change of treatments (e.g., when moving from photon to proton therapy). Such prospective data registration programs are in place at a few leading institutions, with the Dutch‐coordinated effort in head‐and‐neck cancer radiotherapy across modalities and institutions as a prime example (Verdonck‐de Leeuw *et al.*, 2019). Patient‐reported outcomes (PROs) are also of great interest in relation to toxicity assessments, and some data suggest that they can improve routine clinical care (Basch *et al.*, 2017). More research is needed, however, into the relationship that exists between PROs, and physician‐assessed and analytical toxicity endpoints.

In order to be relevant and acceptable to radiation oncologists, predictive models must account for already well‐established risk factors. In terms of the risk of toxicity, several clinical covariates have already been established (Appelt *et al.*, 2014; Thor *et al.*, 2019; Vogelius and Bentzen, 2012), and should be accounted for in the modeling. When it comes to disease‐control endpoints, adjustment for disease stage is clearly necessary (and often available in tumor registries), but more detailed knowledge of disease burden and other prognostic factors might be important as well, in order to test the added utility of novel prognostic assays. For example, tumor volume and HPV status have been shown to provide more robust predictors of survival than radiomics features in head‐and‐neck cancer (Ger *et al.*, 2019). Similar examples exist of clinical factors that confound image‐derived features in lung cancer (Davey *et al.*, 2020). An important general discussion of such problems can be found in Welch *et al. *(2019).

Some authors have argued in favor of registry‐based studies by highlighting the many obstacles that are encountered when designing and conducting large, randomized controlled trials (Quon *et al.*, 2019). The problem remains, however, that even after adjusting for available known covariates, hidden biases might still exist. This is exactly why the randomized controlled trial remains the gold standard of generating medical evidence in comparative effectiveness research. This does not preclude, however, mathematical modeling and data science methods as a component of evidence‐based medicine.

## 4 Mathematical modeling and evidence‐based medicine

Mathematical modeling is an attractive method for data‐driven inference, where evidence from randomized trials is not available, trials are not feasible, or as a tool to describe heterogeneity in treatment effect. But how do we build confidence in a predictive model that supports clinical decisions? A key element to this end is external validation, that is, a quantitative assessment of a model's performance in an independent dataset. There is a rich statistical literature on the validation of models (Collins *et al.*, 2014, 2015). However, the term validation itself is not consistently defined (Altman and Royston, 2000), especially in radiation oncology, where exposure variables are often correlated. Still, most validation studies tend to conclude that the model tested is indeed valid! But there is no consensus among radiation oncology modelers as to what this exactly means. And validation is only meaningful within a carefully specified domain; models are most often not generalizable across domains. For example, a dose–volume–response model developed from photon therapy outcomes in adults may have been ‘validated’ in a similar setting but may not perform well in a cohort of patients treated with proton therapy, or in a population of, say, pediatric patients.

Using the literature, we have compared data science approaches against the gold standard of the randomized controlled trial in some radiotherapy‐relevant cases (Ang *et al.*, 2014; McGale *et al.*, 2016; Pignon *et al.*, 2009; Zandberg *et al.*, 2018), where the effect size of an intervention has been estimated using both methods (Fig. 4). In many cases, the randomized controlled trial and registry‐based effect estimates do not fall on the diagonal line of agreement, even when considering their confidence intervals. Or they are in direct contradiction (as shown by the gray zones in Fig. 4). The meta‐analysis by Pignon *et al. *(2009) shows that the coordinated synthesis of trial outcomes can yield statistical power that is comparable to that of registry analysis but without the associated risk of bias. It is remarkable that the confidence intervals of well‐conducted meta‐analyses are often comparable to those of registry studies, despite fewer patients (Pignon *et al.*, 2009). This is an indication that statistical power in registry studies is not limited by sampling variation alone, but by an overdispersion of effect sizes between individuals in the data, resulting from heterogeneity of treatment effects. It should be emphasized that for registry studies, it is the management of bias/confounding that dominates over statistical sampling uncertainty. *P*‐values, which were developed to compare small sample sizes, often look impressive at face value in registry studies, but should largely be ignored because bias/confounding is the real concern (cf. Fig. 4). Effect sizes with confidence intervals are much more informative, but even these should be tempered by a careful analysis of possible bias.

The limitations of data science should thus be recognized in comparative effectiveness research. However, data science is an important and very relevant supplement to the paradigm of translational research, in particular for questions that are not amenable to being investigated by randomized controlled trials. This includes the previously mentioned example of a dose–response relationship for cardiotoxicity. In the next section, we discuss the dominant methodological development in data science, artificial intelligence, or, more precisely, deep learning (DL) methods.

## 5 Artificial intelligence: Deep learning

Machine learning is a branch of artificial intelligence, in which a mathematical model is built based on a sample dataset, known as the ‘training dataset’. This field of data science is being revolutionized by DL methods, a term that is typically associated with the training of multiple‐layered/deep neural networks. DL techniques are increasingly being explored in radiotherapy, for purposes such as treatment planning, setup, verification, adaptation, or follow‐up in clinics and in research (Meyer *et al.*, 2018; Sahiner *et al.*, 2019). In contrast to methods that combine selected and designed features and classifiers, DL utilizes end‐to‐end representation learning, in which features and their combinations are learned at multiple hierarchies jointly to solve a particular prediction task directly from data. This is one of the reasons for this approach's success. DL most often involves learning from suitably sampled training data with ground‐truth annotation provided by human experts or observed clinical outcome, so‐called supervised learning. Less frequent applications involve semisupervised and even unsupervised learning, in which learning is done by finding inherent structures in the data with limited or no ground truth provided (see Table 1 for a glossary of terms).

**Table 1:** Glossary of data science terms.

Application programming interface (API)	Communication protocol that allows external communication with software or server. In this field, APIs allow researchers to write code (scripts) to query radiotherapy databases to extract features from (large numbers of) individual patients' scan or dosimetry data
Artificial neural networks (ANNs)	An ANN is a network of artificial neurons, connected such that output from a given neuron forms the input to one or more neurons in the next ‘layer’. Passing input data through many successive such layers allows for complex transformations, that is, complex mathematical functions that link a set of inputs to a specific output
Artificial neuron	The artificial neuron is the basic building block of an ANN. It is a mathematical function that takes multiple real‐valued inputs, each of which is multiplied by a weight. These weighted inputs are then summed and put into a so‐called activation function that outputs a real value. The activation function is typically a nonlinear function, for example, a sigmoid function
Deep learning (DL)	A type of learning that uses multiple ANN layers to progressively extract higher level features from the raw input
Federated learning (a.k.a., distributed learning)	This approach entails training a model simultaneously on several datasets that reside on different servers while communicating model data (such as goodness‐of‐fit data and regression coefficients) rather than exchanging the data itself
Generalization error	Generalization errors are calculated by metrics that quantify the amount of error a prediction algorithm makes on a set of previously unseen data
High‐dimensionality datasets	This is a general term used to describe datasets that contain large numbers of features per patient, including genomic data and image features
Machine learning (ML)	The study of how computers learn from data to solve problems. ML is also used to refer to algorithms or systems that learn from data how to solve a task, as opposed to being explicitly programmed how to do so
Multiple‐layered network/deep neural networks	These are neural networks that consist of many layers of neurons between the input and output, such that the output of one layer becomes input for the next
Ontology	Representation, formal naming, and definition of the data in a field of research, examples are tumor characteristics (e.g., UICC staging), organ delineation/naming, dose descriptors, and disease/procedural codes
Record‐and‐verify databases	Databases that were originally invented to document treatments and reduce risk of errors, and that have evolved into complete information systems that contain image data, planned dose matrices, and detailed delivery data. They usually have some sort of application programming interface
Semisupervised learning	Machine learning from input data, where only a subset of input data is paired with output data, that is, an approach that mixes supervised and unsupervised learning
Single‐layer model	This term describes conventional regression models that could be seen to provide a ‘single layer’: In these models, a single mathematical descriptor (e.g., logistic function or Cox model) connects input data to outcome prediction. It is used for illustration here, but it is not an often‐used term
Supervised learning	The task of learning a function that maps an input to an output, based on example input–output pairs. Regression models are examples of this approach
Tall datasets	These are ‘Big data’ datasets where the number of cases (individuals, patients) is much larger than the number of features per case. Examples are population‐based cancer registries or claims databases
Unsupervised learning	This approach finds patterns in datasets without preexisting labels, that is, based solely on the structure of the input data, which is also known as self‐organization. Hierarchical clustering is an example of such a method
Wide datasets	‘Big data’ datasets, in which the number of features (data items) per case is much larger than the number of cases. Examples include data from genomics or proteomics or from medical imaging

Employed DL models can easily fit to both relevant and spurious signals and even to random noise in the training data (Zhang *et al.*, 2016), and the performance or ability to generalize to unseen data is therefore the relevant measure of success. The generalization error of a DL model, given a problem and training data, is something that must be empirically estimated, and occasional failures should be expected and recognized as an inherent property of the DL approach. As DL methods become an integral part of clinical workflow and research, these risks must be properly assessed. It is not enough to assess a DL model's performance on measures, such as average similarity of segmentations and reconstructed images, with ground truths that are often clinically irrelevant. This is because DL methods are surprisingly good at recognizing and replicating even complex signals that often appear in the training data, while potentially failing to recognize obvious but rare signals that occur clinically (Meyer *et al.*, 2018). Estimates of prediction uncertainty in DL models could be used to raise warning flags in such cases. Such estimate prediction techniques include Monte Carlo dropout (Kendall and Gal, 2017), model ensembles (Lakshminarayanan *et al.*, 2017), and variational autoencoders (Hu *et al.*, 2019; Kohl *et al.*, 2018), which function by allowing many different predictions to be generated for each data point. Under the critical assumption that the training set is representative of the underlying true distributions, such methods should be able to convey the variation of predictions to the end user, whether they are a scientist or clinician. However, more work is needed to bridge this critical gap of conveying uncertainty information when using these novel modeling approaches.

A related critique about DL methods is the ‘black‐box’ nature of its output, which can result in a lack of transparency or even interpretability (Vollmer *et al.*, 2020). Because DL methods consist of deep hierarchies of nonlinear functions, whose many parameters are entirely learned from data, understanding their inner workings and predictions is not straightforward. This contrasts with conventional regression models that base their predictions on handcrafted features, and on the statistical significance and the associated effect size (e.g., relative risk or hazard ratio) for each feature, which can be reported to understand the drivers of the model. Understanding why and how DL models work is an active research area, as is conveying such information to the end user (Gilpin *et al.*, 2018). For instance, possible insights into the inner workings of trained DL models can be gained by perturbing inputs and investigating the possible consequences, or by generating heat maps of the importance of a particular part of the input for a given prediction (Ancona *et al.*, 2017; Sahiner *et al.*, 2019).

The major factor that limits the performance of DL methods in medicine is the lack of good‐quality reference data to learn from, thus emphasizing the importance of bridging the gaps in the data sources discussed earlier in the review. The performance of DL models grows with the amount of data in the training set, and although the performance of individual DL models can become saturated after a certain amount of data is included, further improvements can typically be made by extending model architectures (Hestness *et al.*, 2017; Sun *et al.*, 2017). Making public datasets that comprise protected health information is complicated by patient privacy concerns and laws, but has the potential to contribute enormously to advancing the field. The website grand‐challenge.org hosts a large number of challenges and datasets in medical imaging, including the StructSeg2019 segmentation for radiotherapy planning challenge 2019 (Structseg, 2019). However, there is still a limited number of challenges and datasets for radiotherapy applications available.

## 6 Promising solutions and leaps forward

It is clear, that data loss is a primary obstacle to applying data science to radiation oncology, in particular the loss of detailed information about radiation exposure and image data when linking from dose plan to outcome registries. However, promising tools exist to overcome this data loss.

For instance, modern radiotherapy record‐and‐verify systems now provide users with relatively accessible interfaces to enable them to interact with data on a database level, using a so‐called application programming interface (API). APIs are emerging as automation procedures, and they enable the reporting of dose–volume data for available annotated body regions in the dose planning systems (Cai *et al.*, 2019; Cardan *et al.*, 2019). APIs have also been used to link lung exposure to vital status registries (Stervik *et al.*, 2020). While these methods are still limited to structures delineated at the time of treatment planning and stored in the database, it is still a substantial leap forward from datasets that record dates, times, and prescription dose, but not dose distribution (Rubinstein and Warner, 2018).

Another approach to improve the interoperability of distinct datasets is the establishment of large consortia of collaborators. One example is the Early Breast Cancer Trialists' Collaborative Group (EBCTCG), which combines randomized trials data to achieve statistical power comparable to registry studies (Fig. 4). Another successful example is the Radiogenomics Consortium (RGC). Radiogenomics is the scientific study of the link between early or late radiation toxicity and common genetic variations, such as single nucleotide polymorphisms (SNPs). SNPs occur on average once in every 1000 nucleotides in the human genome, which means that there are roughly 4–5 million SNPs in an individual patient's genome. Due to the high dimensionality of SNP datasets, the early literature on SNP predisposing for radiation toxicity was dominated by false‐positive SNPs that were not validated in independent data series (Barnett *et al.*, 2012a). This issue, combined with the inherently large number of covariates that affect the phenotypic presentation and the relatively low prevalence of many single‐nucleotide variants of potential interest, means that this research field requires relatively large sample sizes to achieve sufficient statistical power to detect clinically relevant effect sizes (Barnett *et al.*, 2012a). To this end, RGC was established in 2009 (West and Rosenstein, 2010). It consists of a large volunteer research network, which currently comprises 222 members in 33 countries across 133 institutions. The RGC has successfully identified SNPs involved in radiation toxicity (Andreassen *et al.*, 2016; Kerns *et al.*, 2019), which are of potential value in guiding therapy decisions in individual patients (Bergom *et al.*, 2019).

Federated learning is another collaborative approach but one that removes the need to share protected health information. However, current publications from federated learning collaborations are limited to sample sizes that are smaller than the most prominent consortia (cf. Fig. 3).

**Fig. 5:**
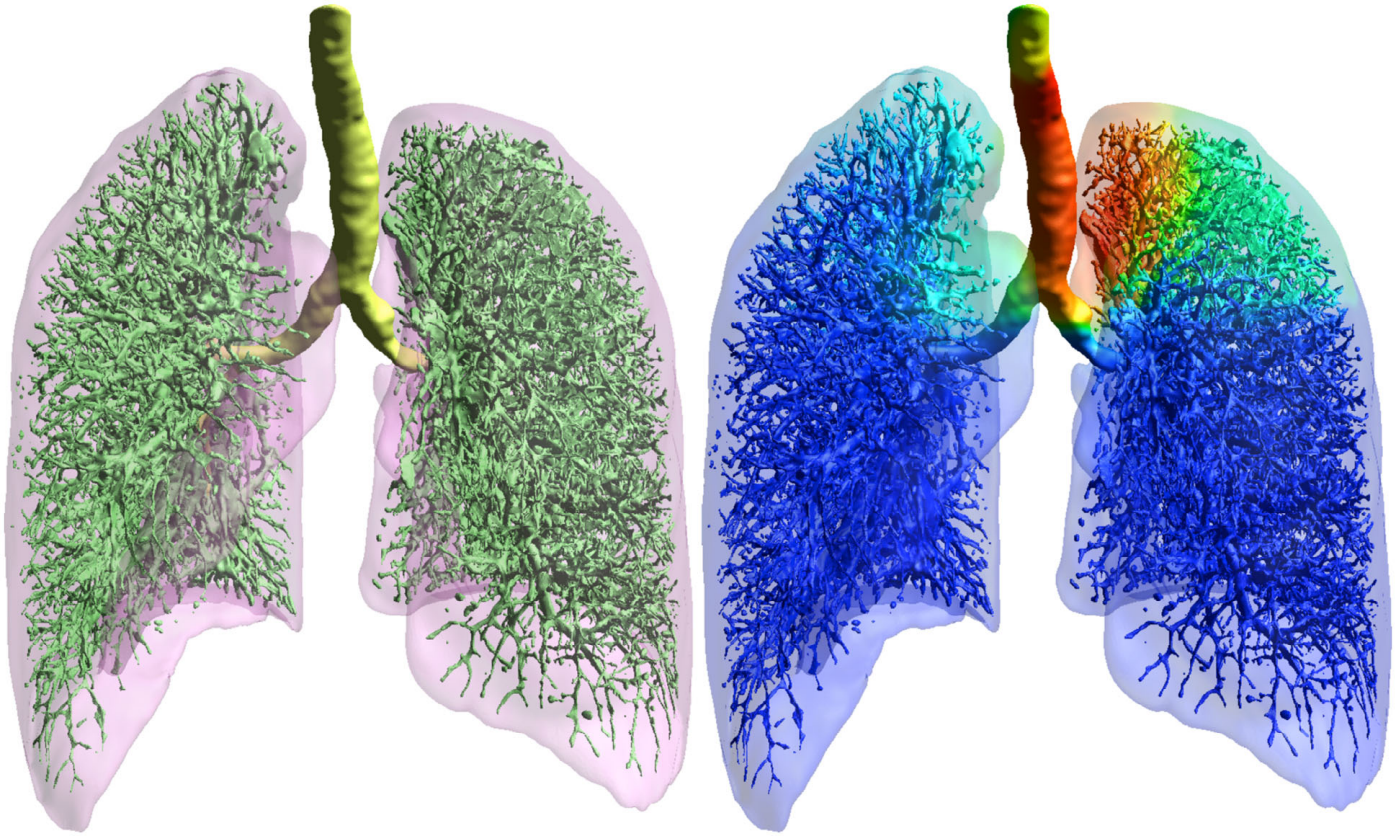
A DL algorithm to analyze radiation exposure in routine clinical setting. Output from a DL algorithm in the form of a 3D U‐Net architecture (Ronneberger *et al.*, 2015). The algorithm was trained on a dataset of manual annotations of lung substructures (vessels and airways). Subsequently, the DL algorithm performed annotations on a previously unseen routine, planning CT scan from a record‐and‐verify system yielding the airway and vessel annotations in green shades depicted on the left. Right: The annotated CT scan is overlaid with a 3D dose color wash to show the potential of automating access to detailed radiotherapy exposure data, which may subsequently be connected to outcome data from the larger registries. Note that some of the smaller vessels are exaggerated in size due to partial volume effects.

Federated learning, consortia, or the automated linkage of record‐and‐verify databases to registries all suffer from a limited ontology of organ structure delineations, as well as limited ontology for procedures and endpoints. Adjusting for between‐data‐series variation is often an important contribution of consortia. For example, RGC has contributed to the field's methodology by providing methods to standardize toxicity scores between data series (Barnett *et al.*, 2012b). The limited availability of organ delineations on routinely treated patients is a challenge that remains, but automated delineation approaches are being actively pursued by both commercial and academic researchers as a way to counteract the continuously increasing burden of substructure delineation in clinical practice (Zhu *et al.*, 2019). These methods are expected to improve in performance in coming years, and can also be used to increase the granularity of organ exposure in all of the above settings. In addition, structure delineation might come to include much more detailed substructures beyond those that are of current relevance for guideline‐driven radiation dose planning. Figure 5 shows an example of automated airway and vessel annotation on a routine scan from record‐and‐verify database, which clearly exceeds the detail that could ever be achieved on substantial patient numbers with manual segmentation.

It should be emphasized that despite the difficulties associated with data linkage and interpretation, a fundamental truth is that the data are there and the data are accessible. We are no longer limited by manual tape switching or by the need to burn and send DVD's, as we were just 10 years ago. We are in an era where it is becoming realistic to perform large‐scale analysis of detailed exposure data across many institutions. Despite the necessary efforts to secure patient confidentiality and legal collaboration agreements, there are good reasons to be optimistic about the ability to harness data science in radiation oncology.

## 7 Towards clinical utility

Turning to the discussion of clinical utility of data science findings (Liu *et al.*, 2019), it should be emphasized that the key word in data science is science rather than data. The success of data science in radiation oncology will ultimately be measured by the clinical utility of the AI tools that are developed or by the novel scientific insights that are uncovered by using such methods, rather than by the simple ability to generate yet another prognostic model or by referring to the amount of data analyzed.

To that end, the methodological challenges must be overcome, and defining analyses and endpoints by availability must be superseded by choosing covariates and endpoints based on clinical relevance and insight. There is an urgent need for cancer‐specific endpoints rather than overall survival, which is used in even the best current tumor registries.

Uncertainty, bias and generalizability should be assessed critically to enable the end user clinician to judge the validity of the model. This is not a new insight (Wyatt and Altman, 1995), but it becomes even more important in the era of neural networks and big data science. The treating physician must fulfill the role of a ‘learned intermediary’ when using decision aids generated by data science and in order to fulfill that role the predictive model must have face validity and provide adequate descriptions of uncertainty. Clinical credibility is certainly still relevant. This is also an educational challenge: Radiation oncologists must be taught how to read, understand, and critically appraise papers that report data science results.

There are two examples of clinically relevant problems that can only be solved by big datasets and where data science is particularly promising: re‐irradiation and detailed pattern‐of‐failure analysis. The retreatment of patients is a very common challenge in radiation oncology, yet our knowledge of organ tolerance is very limited. This is a complex problem where the intensity of the first treatment, the time interval from the first treatment, and the full time line of other anticancer medications are all expected to impact the risk of adverse events. Similarly, our knowledge of the detailed pattern of failure after primary radiation is currently limited to small, opportunistic series that fail to reflect the frequency of cancer recurrence after radiotherapy as a clinical problem (Chao *et al.*, 2003; Due *et al.*, 2014; Nygard *et al.*, 2018). This is a major limiting factor in harnessing the recent technological developments in radiation oncology to provide a dose‐painting approach to radiotherapy, as envisioned decades ago by Ling *et al.* (2000) and Bentzen (2005). Clearly, modern radiotherapy equipment has the ability to meet these needs, but the biological knowledge has to come from data science, and the resulting data‐driven hypotheses for treatment improvements will subsequently need comparative effectiveness testing in controlled clinical trials.

## 8 Conclusion

In conclusion, data science has an important role to play in radiation oncology and we are currently seeing just the first wave of that influence. It should be remembered, however, that data science should be multidisciplinary, and as such, it should involve statistical capabilities, and computational and domain knowledge from clinical radiation oncology.

Clearly, the translational research chain has limitations that are well described in the literature, including the reproducibility crisis and, equally important but less appreciated, the risk of falsely rejecting a good target prior to entering the human testing phase (Baumann *et al.*, 2001; Begley and Ellis, 2012). The addition of big data and data science will supplement, but not replace, the translational research chain. For data science to achieve its potential in radiation oncology, however, several breakthroughs are needed to overcome the limitations mentioned above.

In our view, as shown in Fig. 1, data science should complement translational science and traditional methods should be used to verify conclusions whenever possible. Data science methods applied to large curated datasets linking high‐dimensionality biomarker data with clinical outcome data have the potential to provide tangible benefits to our future patients. Domain knowledge, however, is a key ingredient needed to harness the power of these methods.

## 9 Acknowledgements

The authors acknowledge support from Kirsten and Freddy Johansen's award and National Cancer Institute (Grant No. P30 CA 134274‐04). SMB was supported by funds through the Maryland Department of Health's Cigarette Restitution Fund Program. IRV and JP received research funding from Varian Medical Systems.

## Conflict of interest

The authors declare no conflict of interest.
